# *Helicobacter pylori* seropositivity is associated with antinuclear antibodies in US adults, NHANES 1999–2000

**DOI:** 10.1017/S0950268820000126

**Published:** 2020-02-05

**Authors:** H. C. S. Meier, F. W. Miller, G. E. Dinse, C. R. Weinberg, C. C. Cho, C. G. Parks

**Affiliations:** 1 Joseph J. Zilber School of Public Health, University of Wisconsin-Milwaukee, Milwaukee, WI, USA; 2 Clinical Research Branch, National Institute of Environmental Health Sciences, National Institutes of Health, Durham, NC, USA; 3 Social & Scientific Systems, Durham, NC, USA; 4 Biostatistics and Computational Biology Branch, National Institute of Environmental Health Sciences, National Institutes of Health, Durham, NC, USA; 5 Center for Aging and Translational Research, College of Health Sciences, University of Wisconsin-Milwaukee, Milwaukee, WI, USA; 6 Epidemiology Branch, National Institute of Environmental Health Sciences, National Institutes of Health, Durham, NC, USA

**Keywords:** Antinuclear antibodies, autoimmunity, epidemiology, *H. pylori*

## Abstract

Infectious diseases, such as *Helicobacter pylori*, which produce systemic inflammation may be one key factor in the onset of autoimmunity. The association between *H. pylori* and antinuclear antibodies (ANA), a marker of autoimmunity, has been understudied. Data from the 1999–2000 National Health and Nutrition Examination Survey were used to evaluate the cross-sectional association between *H. pylori* seroprevalence and ANA positivity in US adults aged ≥20 years. ANA was measured in a 1:80 dilution of sera by indirect immunofluorescence using HEp-2 cells (positive ⩾3). *H. pylori* immunoglobulin G enzyme-linked immunosorbent assays were used to categorise individuals as seropositive or seronegative. *H. pylori* seropositivity and ANA positivity were common in the adult US population, with estimated prevalences of 33.3% and 9.9%, respectively. Both were associated with increasing age. *H. pylori* seropositivity was associated with higher odds of ANA (prevalence odds ratio = 1.89, 95% confidence interval = 1.08–3.33), adjusted for age, sex, race/ethnicity, educational attainment and body mass index. *H. pylori* infection may be one key factor in the loss of self-tolerance, contributing to immune dysfunction.

## Introduction

Loss of self-tolerance is a hallmark of autoimmune disease, but less well understood are the drivers of self-tolerance loss in the immune system. Antinuclear antibodies (ANA), representing humoral immunity to cellular components, are one indicator of autoimmunity that occurs in the context of the failure of self-tolerance mechanisms. ANA are a feature of Systemic Lupus Erythematosus [1] and other systemic autoimmune diseases. The prevalence of ANA in the general population ranges from 12% to 16%, is higher in women, and increases with age [2].

Autoimmunity is thought to result from the interactions of environmental and genetic risk factors [3, 4]. Infectious agents may be important environmental exposures resulting in autoimmunity. In particular, chronic infections that have evolved extensive immune evasion mechanisms are of interest as potential drivers of self-tolerance loss. One such infectious agent is *Helicobacter pylori*, a bacterium often contracted in childhood, which colonises the mucosal layer of the gastric epithelium [3]. *H. pylori* infection is common, with the prevalence being approximately 50% worldwide [5, 6], and is well-known for its causative role in gastritis and peptic ulcer disease [7]. Importantly, *H. pylori* has evolved a variety of immune evasion tactics, including circumventing recognition by the innate immune system, inhibition of phagocytic killing, modulation of antigen-presenting cell functions and manipulation of host T-cell responses [8]. Without antibiotic treatment, *H. pylori* infection may remain for many years, if not the entire life of the individual, as the infection is often asymptomatic [7].

Previous studies have examined the role of *H. pylori* in autoimmune disease due to its common occurrence and influence on the immune system [3]. However, the relationship between *H. pylori* and ANA in the general population is unknown. Using data from the 1999–2000 National Health and Nutrition Examination Survey (NHANES), we evaluated the cross-sectional association between *H. pylori* seroprevalence and ANA positivity in the adult US population.

## Materials and methods

### Study population

Data were from NHANES, which is a multistage, nationally representative survey sample of the non-institutionalised US population [9]. The NHANES protocol was approved by the human subjects Institutional Review Board of the US Centers for Disease Control and Prevention, and written informed consent was obtained from all participants. Only the 1999–2000 NHANES cycle had existing laboratory data on both *H. pylori* seropositivity and ANA. Individuals with information on laboratory and demographic covariates of interest were included in this study, resulting in a final sample size of 1005 adults aged 20 years or older.

### *H. pylori* seropositivity

As detailed in the NHANES protocol [10], serum samples from NHANES participants (*N* = 7493) were collected via venepuncture and stored at −80 °Celsius until they were tested at the University of Washington. *H. pylori*-specific immunoglobulin G (IgG) was measured using Wampole Laboratories (Cranbury, NJ) *H. Pylori* IgG Enzyme-Linked Immunosorbent Assay (ELISA). Standard ELISA cut-offs were used to categorise participants into seropositive (optical density (OD) value ≥1.1) or seronegative (OD value <0.9) to *H. pylori*. Equivocal values (0.9–1.1) were categorised as seronegative to produce conservative estimates of serostatus.

### ANA positivity

Autoantibody testing was previously conducted on stored sera from a representative subsample (*N* = 4754) of survey participants [11]. Standard indirect immunofluorescence was used to measure ANA in serum specimens, based on commercial HEp-2 ANA slides (Inova Diagnostics) with 1:80 dilutions of sera and staining with DyLight 488-conjugated donkey anti-human IgG antibodies (Jackson ImmunoResearch), as previously reported [2]. Staining intensities were graded from 0 to 4 relative to a standard reference gallery, with values of 3 and 4 considered indicative of ANA positivity [2]. Two independent raters agreed on >95% of the readings for overall intensity ratings, and differences were resolved by consensus or adjudicated by a third reviewer.

### Covariates

Covariates considered for this analysis include age, sex, race/ethnicity, educational attainment and body mass index (BMI). These covariates were chosen based on *a priori* knowledge of their relationships with both *H. pylori* serostatus and ANA positivity. Age was measured in years and categorised into approximate tertiles (20–34 years, 35–59 years and 60 years or older) for age-stratified analyses. Sex was dichotomised into male or female. Race was categorised into non-Hispanic White, non-Hispanic Black or Other. Educational attainment was used as a proxy for socioeconomic status as it is established early in life, not modified by chronic disease and contributes to the development of health capital [12, 13]. Educational attainment was categorised as less than high school, high school or more than high school. BMI was calculated by dividing the weight in kg by the height in m^2^ and then classified as normal (<25 kg/m^2^), overweight (25 to <30 kg/m^2^) or obese (≥30 kg/m^2^). A dichotomous variable representing medical history of ulcers was ascertained by asking participants ‘Have you ever been told by a doctor or other health professional that you had an ulcer (stomach, duodenal or peptic)?’ Current use of omeprazole, lansoprazole, rabeprazole, pantoprazole, esomeprazole or dexlansoprazole was coded as using *vs.* not using proton pump inhibitor medication. No individuals reported taking *H. pylori* eradication agents within the last month, including bismuth subsalicylate, metronidazole, tetracycline, amoxicillin and/or clarithromycin. Individuals with anti-extractable nuclear antigen (ENA) antibodies (measured using previously described immunoprecipitation methods among the ANA positive [2]) or self-reported autoimmune disease (thyroid problems, rheumatoid arthritis or type 1 diabetes) were classified as having possible autoimmune disease [14].

### Statistical analyses

Analyses were conducted with SAS version 9.4 (SAS Institute, Inc., Cary, NC) using SURVEY procedures and the Taylor series variance estimation to account for the complex survey design. Medical exam unit sampling weights were revised for participation in the substudy as previously described [2]. Bivariate relationships between ANA status, *H. pylori* seropositivity and covariates were assessed using design-based Rao–Scott *χ*^2^ statistics [15]. The association between *H. pylori* seropositivity and ANA was modelled using multiple logistic regression. The continuous age-adjusted prevalence odds ratio (POR) and 95% confidence interval (CI) were estimated first, followed by estimates that also adjusted for sex and race/ethnicity. Finally, a third set of estimates additionally adjusted for educational attainment and BMI. Several sensitivity analyses were performed, including an examination of the association between *H. pylori* and ANA within strata specified by age or sex, as well as one that additionally adjusted for current use of proton pump inhibitors, medical history of ulcer or likely autoimmune disease.

## Results

**Fig. 1.**
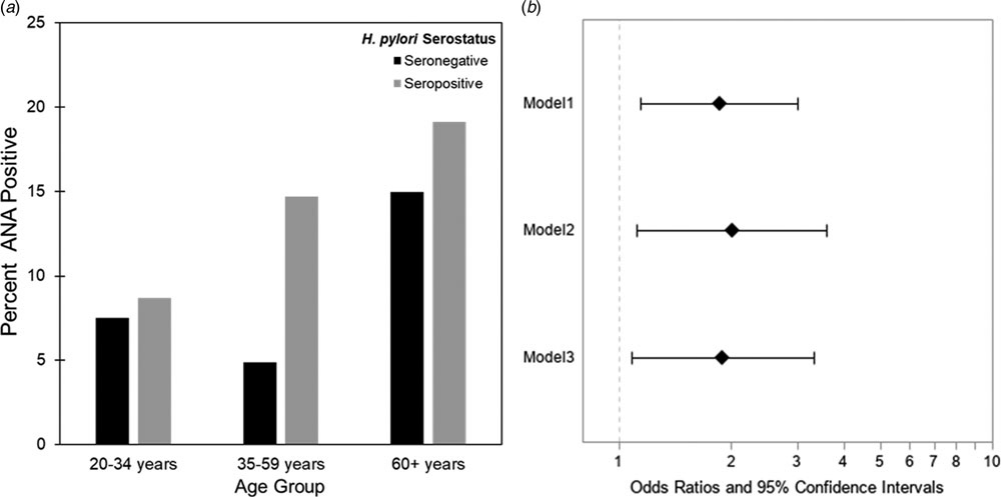
(a) Weighted percent of participants who were ANA positive by age group among *H. pylori* seropositives and seronegatives, NHANES 1999–2000 (*N* = 1005). (b) Association between *H. pylori* seropositivity and ANA positivity, ages 20+, NHANES 1999–2000, *N* = 1005. Model 1 adjusted for age; Model 2 adjusted for age, sex and race/ethnicity; Model 3 adjusted for age, sex, race/ethnicity, BMI and education. Additional adjustment for peptic ulcer, proton pump inhibitor medication use and ENA or autoimmune illness did not meaningfully alter results.

The estimated weighted prevalence of ANA positivity was 9.9% in the adult population and the proportion seropositive for *H. pylori* was 33.3%. The weighted mean age was 45.5 years (range 20–85 years) and 49.8% were female. A majority reported non-Hispanic White race/ethnicity (71%), some college education or above (51%) and normal BMI (38%). Ten percent of participants indicated ever having an ulcer and of those, 21% reported the occurrence of an ulcer in the past 12 months. Prevalence of ANA varied by sex and age, with females and older individuals more likely to be ANA positive, as expected (Table 1). Higher seroprevalence of *H. pylori* was observed with increasing age, and among minorities, those with less than high school educational attainment, and those who self-reported medical history of ulcer (Table 1). The rising prevalence of ANA positivity among *H. pylori* seropositive individuals by age group is depicted in Figure 1a. The greatest difference of ANA positivity by serostatus was observed in the 35–59-year-old age group.

**Table 1.** Characteristics and weighted proportions by ANA status and *H. pylori* serostatus in the adult US population 20+, NHANES 1999–2000

		ANA status			*H. pylori* Serostatus		
	Overall	Negative	Positive		Negative	Positive	
Characteristic	*n* (%)	*n* (%)	*n* (%)	*P*-value^a^	*n* (%)	*n* (%)	*P*-value^a^
	1005	899 (90.1)	116 (9.9)		541 (66.7)	464 (33.3)	
Sex				<0.0001			0.59
Female	503 (49.8)	421 (84.7)	82 (15.3)		277 (65.8)	226 (34.2)	
Male	502 (50.2)	468 (95.4)	34 (4.6)		264 (67.5)	238 (32.5)	
Age (years)				0.009			0.004
20–34	251 (30.6)	226 (92.2)	25 (7.8)		171 (75.4)	80 (24.6)	
35–59	393 (47.5)	354 (91.8)	39 (8.2)		202 (65.6)	191 (34.4)	
60+	361 (21.9)	309 (83.3)	52 (16.7)		168 (56.9)	193 (43.1)	
Race/ethnicity				0.78			<0.0001
Non-Hispanic White	461 (71.1)	412 (90.1)	49 (9.9)		337 (76.6)	124 (23.4)	
Non-Hispanic Black	167 (9.1)	148 (87.9)	19 (12.1)		78 (50.4)	89 (49.6)	
Other	377 (19.8)	329 (90.7)	48 (9.3)		126 (38.5)	251 (61.5)	
BMI (kg/m^2^)				0.29			0.40
Normal (<25)	340 (38.1)	291 (88.3)	49 (11.7)		191 (66.6)	149 (33.4)	
Overweight (25 to <30)	363 (33.7)	328 (89.9)	35 (10.1)		190 (64.2)	173 (35.8)	
Obese (30+)	302 (28.2)	270 (92.5)	32 (7.5)		160 (69.7)	142 (30.3)	
Education				0.04			<0.0001
Less than high school	382 (22.9)	338 (90.2)	44 (9.8)		137 (47.8)	245 (52.2)	
High school	221 (25.7)	189 (85.8)	32 (14.2)		131 (67.6)	90 (32.4)	
More than high school	402 (51.4)	362 (92.1)	40 (7.9)		273 (74.6)	129 (25.4)	
Ever had ulcer (stomach, duodenal or peptic), *N* = 1004				0.64			0.03
No	903 (89.8)	797 (90.2)	106 (9.8)		492 (68.0)	411 (32.0)	
Yes	101 (10.2)	91 (80.6)	10 (11.4)		48 (55.0)	53 (45.0)	
Proton pump inhibitor medication, *N* = 997				0.20			0.45
No	952 (95.6)	843 (90.3)	109 (9.7)		513 (67.1)	439 (32.9)	
Yes	45 (4.4)	39 (84.1)	6 (15.9)		23 (59.7)	22 (40.3)	

a Rao–Scott *χ*^2^.

Results from multiple logistic regressions are provided in Figure 1b. *H. pylori* seropositive individuals had almost twice the age-adjusted odds of ANA positivity as *H. pylori* seronegative individuals (POR: 1.88, 95% CI 1.19–2.99). This association was robust to adjustment for sex, race/ethnicity, educational attainment and BMI (POR: 1.99, 95% CI 1.18–3.35). When stratified by sex (Supplementary Table S1) or age (Supplementary Table S2), the association between *H. pylori* and ANA appeared to be stronger in males (*P*-value interaction: 0.11) and in the 35–59-year-old age group. Sensitivity analyses adjusting for proton pump inhibitor medication use, self-reported history of an ulcer or likely autoimmune disease (ENA or autoimmune illness) did not meaningfully alter results.

## Discussion

In this cross-sectional study of a sample of the general US population, we found that *H. pylori* seropositivity was associated with ANA. Previous studies have documented the presence of potentially damaging autoantibodies to self-epitopes in response to *H. pylori* infection [8], including anti-platelet autoantibodies in immune thrombocytopenia (ITP) [16], anti-parietal cell autoantibodies present in the stomach [17] and anti-gastric autoantibodies in sera [18]. In exploring infectious determinants of autoimmune disease, research has shown an association between *H. pylori* and ANA in Europe and Latin America [19]. Here we extend this analysis to the US population.

*H. pylori* has evolved extensive mechanisms to evade recognition by the immune system as well as to establish persistent infection through manipulating the innate and adaptive immune systems [8]. The long-term consequences of immune evasion, suppression and induction activities by *H. pylori* beyond gastric health effects are still being uncovered. Many of the negative health outcomes associated with *H. pylori* are thought to be a consequence of systemic effects produced by the chronic inflammatory state triggered by this infection [8]. *H. pylori* may play a pathogenic role in the development of autoimmunity and autoimmune disease [3] as its immune evasion mechanisms have been shown to induce autoantibodies, likely through inflammatory mechanisms [20]. For example, *H. pylori* upregulates interferon (INF)-*γ*, a cytokine that mediates the host response to bacterial infection and autoimmune disease [21]. INF-*γ* treatment for cancer has been shown to induce ANA [22], and upregulation of INF-*γ* by *H. pylori* could potentially induce ANA positivity.

In our study, the 35–59-year-old age group exhibited the strongest association between *H. pylori* seropositivity and ANA. Previous studies of age trends in ANA positivity have posited that differential exposure to factors related to ANA development by age may be driving non-linear trends [2]. In this study, the 35–59-year-old age group made up the greatest proportion of individuals who were *H. pylori* seropositive. This age range may represent a critical point in the life course where individuals have lived long enough to not only be exposed to *H. pylori*, but also experience pathophysiological consequences of the infection, such as systemic inflammation and other downstream health effects.

Sensitivity analyses also revealed that the association between *H. pylori* and ANA may be stronger in men than in women; however, the analysis was underpowered due to relatively few ANA seropositive males. Further studies of sex-specific effects are needed to better understand important differences in ANA and other autoimmunity markers by sex.

A previous study showed omeprazole use, a common proton pump inhibitor medication for ulcers, was associated with higher risk of ANA positivity [14]. Prior to this study, omeprazole was not thought to induce autoimmunity nor was it linked with autoimmune disease. The association of *H. pylori* and ANA in the present study was robust to adjustment for proton pump inhibitor medication use (POR = 2.05, 95% CI 1.19–3.54), therefore, it is possible omeprazole use was a proxy for *H. pylori* infection in the previous analysis of medications and ANA.

Our study has several limitations, including the exclusion of institutionalised adults by NHANES and the cross-sectional study design. We do not know the timing of *H. pylori* infection nor do we know the onset date of ANA. However, *H. pylori* is often acquired in childhood [5] when ANA prevalence is the lowest [2] so it is likely that *H. pylori* infection preceded ANA positivity. Another limitation is that we do not know if individuals have active or previous *H. pylori* infection, only that individuals have been exposed to *H. pylori* and mounted an immune response. Importantly, no individuals reported taking *H. pylori* eradication medications in the last month, meaning that *H. pylori* exposure had at least a 1-month lag time to induce an inflammatory response before ANA assessment. Future research with longer medication ascertainment and longitudinal measurement of both *H. pylori* and ANA is needed to clarify the temporal relationship, as well as better understand whether ANA are transient or permanent, especially in relation to treatment with *H. pylori* eradication medication. For example, studies of ITP have shown that *H. pylori* eradication treatment results in the resolution of autoimmune disease [23, 24]. If future research supports a causal association between *H. pylori* and ANA, screening for *H. pylori* and treatment of the infection may play a role in reducing autoimmunity and potentially prevent progression to autoimmune disease. Finally, we are limited to data from 1999–2000 as both ANA and *H. pylori* have not been measured in more recent NHANES cycles; however, the results remain relevant as a previous study demonstrated *H. pylori* seroprevalence levels were consistent over 10 years from NHANES III to 1999–2000 [5] and although treatment for *H. pylori* infection is available, many individuals do not experience symptoms and do not seek treatment, indicating *H. pylori* seroprevalence is unlikely to have changed meaningfully in the USA over time.

Despite these limitations, our study extends previous research on the association of *H. pylori* and ANA to a large, representative sample of the adult US population. We observed a strong and biologically plausible association that was robust to adjustment for multiple factors. Not fully understanding the drivers of autoimmunity is a key research gap. Infectious diseases, such as *H. pylori*, that produce systemic inflammation may be one key factor in the loss of self-tolerance. Future work exploring the enduring consequences of immune evasion and suppression activities by *H. pylori* over the life course is warranted. Specifically, future research should examine possible mechanisms by which altered immune function resulting from persistent infection, including autoimmunity and inflammation, influence subsequent chronic disease aetiology and progression.

## Supplementary material

For supplementary material accompanying this paper visit https://doi.org/10.1017/S0950268820000126. click here to view supplementary material

## References

[1] Arbuckle MR (2003) Development of autoantibodies before the clinical onset of systemic lupus erythematosus. The New England Journal of Medicine 349, 1526–1533. 14561795 10.1056/NEJMoa021933

[2] Satoh M (2012) Prevalence and sociodemographic correlates of antinuclear antibodies in the United States. Arthritis and Rheumatism 64, 2319–2327. 22237992 10.1002/art.34380 PMC3330150

[3] Hasni SA (2012) Role of *Helicobacter pylori* infection in autoimmune diseases. Current Opinion in Rheumatology 24, 429–434. 22617822 10.1097/BOR.0b013e3283542d0b PMC3643302

[4] Miller FW (2011) Environmental agents and autoimmune diseases. Advances in Experimental Medicine and Biology 711, 61–81. 21627043 10.1007/978-1-4419-8216-2_6

[5] Grad YH, Lipsitch M and Aiello AE (2012) Secular trends in *Helicobacter pylori* seroprevalence in adults in the United States: evidence for sustained race/ethnic disparities. American Journal of Epidemiology 175, 54–59. 22085628 10.1093/aje/kwr288 PMC3244610

[6] Pounder RE and Ng D (1995) The prevalence of *Helicobacter pylori* infection in different countries. Alimentary Pharmacology & Therapeutics 9(suppl. 2), 33–39. 8547526

[7] McColl KE (2010) Clinical practice. *Helicobacter pylori* infection. The New England Journal of Medicine 362, 1597–1604. 20427808 10.1056/NEJMcp1001110

[8] Lina TT (2014) Immune evasion strategies used by *Helicobacter pylori*. World Journal of Gastroenterology 20, 12753–12766. 25278676 10.3748/wjg.v20.i36.12753 PMC4177461

[9] Curtin LR (2012) The National Health and Nutrition Examination Survey: sample design, 1999–2006. Vital Health Statistics 2, 1–39. 22788053

[10] Centers for Disease Control and Prevention. National Health and Nutrition Examination Survey 1999–2001 Data Documentation, Codebook, and Frequencies – *H. pylori* Available at https://wwwn.cdc.gov/Nchs/Nhanes/1999-2000/LAB11.htm (Accessed 5 November 2018).

[11] Centers for Disease Control and Prevention. National Health and Nutrition Examination Survey 1999–2001 Data Documentation, Codebook, and Frequencies: Autoantibodies – Immunofluorescence & Immunoprecipitation Analyses. Available at https://wwwn.cdc.gov/Nchs/Nhanes/1999-2000/SSANA_A.htm (Accessed 5 November 2018).

[12] Meier HCS (2016) Early life socioeconomic position and immune response to persistent infections among elderly Latinos. Social Science & Medicine 166, 77–85. 27543684 10.1016/j.socscimed.2016.07.004 PMC5573138

[13] Bobak M (2000) Own education, current conditions, parental material circumstances, and risk of myocardial infarction in a former communist country. Journal of Epidemiology and Community Health 54, 91–96. 10715740 10.1136/jech.54.2.91 PMC1731618

[14] Dinse GE (2018) Prescription medication use and antinuclear antibodies in the United States, 1999–2004. Journal of Autoimmunity 92, 93–103. 29779929 10.1016/j.jaut.2018.05.006 PMC6054905

[15] Rao JNK and Scott AJ (1987) On simple adjustments to chi-squared tests with survey data. Annals of Statistics 5, 385–397.

[16] Kuwana M (2014) *Helicobacter pylori*-associated immune thrombocytopenia: clinical features and pathogenic mechanisms. World Journal of Gastroenterology 20, 714–723. 24574745 10.3748/wjg.v20.i3.714 PMC3921481

[17] Basso D (2000) Antigastric autoantibodies in *Helicobacter pylori* infection: role in gastric mucosal inflammation. International Journal of Clinical Laboratory Research 30, 173–178. 11289707 10.1007/s005990070003

[18] Faller G (2000) Mucosal production of antigastric autoantibodies in *Helicobacter pylori* gastritis. Helicobacter 5, 129–134. 10971676 10.1046/j.1523-5378.2000.00020.x

[19] Ram M (2013) *Helicobacter pylori* serology in autoimmune diseases – fact or fiction? Clinical Chemistry and Laboratory Medicine 51, 1075–1082. 23079514 10.1515/cclm-2012-0477

[20] Amedei A (2003) Molecular mimicry between *Helicobacter pylori* antigens and H^+^, K^+^–adenosine triphosphatase in human gastric autoimmunity. Journal of Experimental Medicine 198, 1147–1156. 14568977 10.1084/jem.20030530 PMC2194239

[21] Alvarez-Arellano L and Maldonado-Bernal C (2014) *Helicobacter pylori* and neurological diseases: married by the laws of inflammation. World Journal of Gastrointestinal Pathophysiology 5, 400–404. 25400983 10.4291/wjgp.v5.i4.400 PMC4231504

[22] Wandl UB (1992) Lupus-like autoimmune disease induced by interferon therapy for myeloproliferative disorders. Clinical Immunology and Immunopathology 65, 70–74. 1382910 10.1016/0090-1229(92)90250-r

[23] Frydman GH (2015) *Helicobacter pylori* eradication in patients with immune thrombocytopenic purpura: a review and the role of biogeography. Helicobacter 20, 239–251. 25728540 10.1111/hel.12200 PMC4506733

[24] Vanegas YAM and Vishnu P (2019) Management of *Helicobacter pylori* in patients with immune thrombocytopenia. Hamostaseologie 39, 279–283. 30891714 10.1055/s-0039-1683974

