# Supplementary material for: Helicobacter pylori seropositivity is associated with antinuclear antibodies in US adults, NHANES 1999–2000
Source: Epidemiol Infect. 2020 Feb 5;148:e20. doi: 10.1017/S0950268820000126 (PMC7019483; doi:10.1017/S0950268820000126)
Supplement: Supplementary file 1 [file S0950268820000126sup001.zip › S0950268820000126sup001/Meier_Supplemental_Table1.docx]

**Supplemental Table 1.** Sex-stratified association between H. pylori seropositivity and ANA, 20+, NHANES 1999-2000, N=1005

| FEMALE (N=503) | |  |  | MALE (N=502) | |  |
| --- | --- | --- | --- | --- | --- | --- |
|  | Odds Ratio ANA | (95% CI) |  |  | Odds Ratio ANA | (95% CI) |
| Model 1 | 1.50 | (0.89, 2.53) |  | Model 1 | 4.03 | (1.22, 13.37) |
| Model 2 | 1.67 | (0.89, 3.13) |  | Model 2 | 3.98 | (1.14, 13.81) |
| Model 3 | 1.54 | (0.86, 2.75) |  | Model 3 | 4.24 | (1.06, 16.99) |
| Model 4 | 1.59 | (0.90, 2.82) |  | Model 4 | 4.01 | (1.14, 14.09) |
| Model 5 | 1.61 | (0.86, 3.00) |  | Model 5 | 4.23 | (1.06, 16.93) |

Model 1: unadjusted

Model 2: adjusted for sex and race

Model 3: adjusted for sex, race, BMI and education;

Model 4: adjusted for race, BMI, education and ever having an ulcer

Model 5: adjusted for race, BMI, education and proton pump inhibitor use

95% CI: 95% confidence interval
