# Supplementary material for: Helicobacter pylori seropositivity is associated with antinuclear antibodies in US adults, NHANES 1999–2000
Source: Epidemiol Infect. 2020 Feb 5;148:e20. doi: 10.1017/S0950268820000126 (PMC7019483; doi:10.1017/S0950268820000126)
Supplement: Supplementary file 1 [file S0950268820000126sup001.zip › S0950268820000126sup001/Meier_Supplemental_Table2.docx]

**Supplemental Table 2.** Age-stratified association between H. pylori seropositivity and ANA, 20+, NHANES 1999-2000, N=1005

|  | Age 20-35 (N=251) | | |  | Age 35-59 (N=393) | | |  | Age 60+ (N=361) | | |
| --- | --- | --- | --- | --- | --- | --- | --- | --- | --- | --- | --- |
|  |  | 95% CI | |  |  | 95% CI | |  |  | 95% CI | |
|  | OR | LCL | UCL |  | OR | LCL | UCL |  | OR | LCL | UCL |
| Model 1 | 1.16 | 0.48 | 2.81 |  | 3.37 | 1.30 | 8.74 |  | 1.35 | 0.54 | 3.34 |
| Model 2 | 1.21 | 0.60 | 2.43 |  | 3.81 | 1.37 | 10.57 |  | 1.62 | 0.59 | 4.41 |
| Model 3 | 1.10 | 0.53 | 2.28 |  | 3.52 | 1.36 | 9.13 |  | 1.69 | 0.64 | 4.47 |
| Model 4 | 1.50 | 0.66 | 3.42 |  | 3.47 | 1.36 | 8.84 |  | 1.60 | 0.61 | 4.21 |
| Model 5 | 1.59 | 0.69 | 3.70 |  | 3.85 | 1.50 | 9.90 |  | 1.72 | 0.58 | 5.10 |

Model 1: unadjusted

Model 2: adjusted for sex and race

Model 3: adjusted for sex, race, BMI and education;

Model 4: adjusted for race, BMI, education and ever having an ulcer

Model 5: adjusted for race, BMI, education and proton pump inhibitor use

OR: odds ratio for ANA; 95% CI: 95% confidence interval
